# Complete Genome Sequences of Clinical Pandoraea fibrosis Isolates

**DOI:** 10.1128/MRA.00060-20

**Published:** 2020-03-26

**Authors:** Miranda E. Pitt, Son H. Nguyen, Tânia P. S. Duarte, Louise F. Roddam, Mark A. T. Blaskovich, Matthew A. Cooper, Lachlan J. M. Coin

**Affiliations:** a Institute for Molecular Bioscience, The University of Queensland, Brisbane, Queensland, Australia; b The Peter Doherty Institute for Infection and Immunity, The University of Melbourne, Melbourne, Victoria, Australia; c School of Medicine, University of Tasmania, Hobart, Tasmania, Australia; University of Arizona

## Abstract

Pandoraea fibrosis is a newly identified Gram-negative bacterial species that was isolated from the respiratory tract of an Australian cystic fibrosis patient. The complete assembled genome sequences of two consecutive isolates (second isolate collected 11 months after antibiotic treatment) from the same individual are presented here.

## ANNOUNCEMENT

*Pandoraea* species have been isolated from cystic fibrosis (CF) airways, lung/blood from non-CF individuals, and environmental (e.g., soil and water) sources (1–6). Clinical isolates are predominantly multidrug resistant (MDR) and commonly cocolonize with Pseudomonas aeruginosa when recovered from CF sputum (4, 7). *Pandoraea* spp. can establish chronic airway infections and cause significant lung function deterioration, possibly due to eliciting a proinflammatory response (7–11). The detection of *Pandoraea* spp. remains difficult (misidentified as Burkholderia cepacia or a *Ralstonia* sp.); hence, the prevalence of *Pandoraea* sp.-associated infections may be underreported (1, 12, 13). To date, 11 *Pandoraea* species have been described, including the recently characterized species Pandoraea fibrosis (14).

Only three incomplete *P. fibrosis* genome sequences have been reported (NCBI), of which two were collected from a CF patient admitted to the Royal Hobart Hospital (Tasmania, Australia) (14, 15). These isolates include the designated strain 6399^T^ and a subsequent isolate (7641) collected 11 months after antibiotic treatment. These MDR strains, initially detected as Pandoraea apista and then Pandoraea pnomenusa, recently underwent polyphasic taxonomic analysis and were identified as *P. fibrosis* (14–16). Given the overall dearth of complete clinical *Pandoraea* genomes, combined with our limited understanding of virulence and resistance mechanisms of *Pandoraea* spp., we sought to complete these two *P. fibrosis* genomes and identify genes associated with disease.

Strains 6399^T^ and 7641 were previously recovered from CF sputa (14, 15) via growth on Burkholderia cepacia-selective medium and stored at −80°C in lysogeny broth (LB) supplemented with 20% (vol/vol) glycerol. Glycerol stocks were streaked onto LB agar for colony isolation and grown in LB. Subsequently, high-molecular-weight (HMW) DNA was purified from 6399^T^ using the DNeasy blood and tissue kit (Qiagen) and a >8-kb size selection performed via the BluePippin instrument (Millennium Science), while 7641 was prepared using the MagAttract HMW kit (Qiagen). DNA extracts underwent Oxford Nanopore Technologies (ONT) MinION sequencing with the SQK-LSK108_1D long reads (6399^T^) or the RAD003-v2 (7641) kit and were sequenced on R9.4 flowcells. Sequences were base called using Albacore 2.3.1, which yielded 33× (6399^T^) and 13× (7641) coverage, respectively.

Genomes were assembled using both Illumina MiSeq data (16) and ONT reads via Unicycler v0.3.7 (17). Default parameters were used for all software unless otherwise noted. The completed genomes were 5,592,065 bp (6399^T^) and 5,592,064 bp (7641), with both exhibiting a G+C content of 62.8%. Similar to the prior Illumina-only assembly (16), genomes were annotated using the Rapid Annotations using Subsystem Technology tool kit (RASTtk) v2.0 with taxonomy identity 93220 (*P. pnomenusa*) (18). The RASTtk pipeline identified a total of 5,103 (6399^T^) and 5,104 (7641) protein-coding sequences (57 genes associated with virulence, disease, and defense and 42 genes linked to antibiotic resistance and toxic compounds), with both isolates containing 76 RNA genes (64 tRNA genes). Gene content was similar between the two isolates, although the iron acquisition gene *ybtA* was absent in 7641. A comparison to *P. pnomenusa* RB38 (19) revealed that 6399^T^ has 280 genes unique to *P. fibrosis*. Conversely, the Prokaryotic Genome Annotation Pipeline identified 4,801 (6399^T^) and 4,799 (7641) protein-coding sequences and 81 RNA genes (65 tRNA genes) (20). ResFinder 3.2 identified the *bla*_OXA-153_ gene (86.21% identity; beta-lactam resistance) in both strains (21). Compared with 6399^T^, 7641 harbored 3 indels, 3 missense mutations, and 1 large 1,451,403-bp inversion (BWA-MEM, GATK, and snpEff) (22–24). The completion of these genome sequences has provided insight into *P. fibrosis* virulence and antibiotic resistance mechanisms.

### Data availability.

This whole-genome sequencing project has been deposited in DDBJ/EMBL/GenBank under accession numbers CP047385 for strain 6399 (BioProject number PRJNA266749, BioSample number SAMN03174139, and SRA numbers SRX7812756 [Illumina reads] and SRX6578695 [ONT reads, fast5]) and CP047386 for strain 7641 (BioProject number PRJNA266765, BioSample number SAMN03174414, and SRA numbers SRX7812757 [Illumina reads] and SRX6578757 [ONT reads, fast5]). The assembly versions described in this paper are the second versions.
